# Treatment of aortic infections with allografts: 20 years of experience of the European Homograft Bank (EHB) in Brussels

**DOI:** 10.1186/1749-8090-8-S1-O105

**Published:** 2013-09-11

**Authors:** Ramadan Jashari

**Affiliations:** 1 European Homograft Bank (EHB) International Association, Brussels, Belgium

## Aim

Infection of native arteries and/or prosthetic material is one the most frequent indications to use cryopreserved arterial homografts. For more than 20 years the European Homograft Bank (EHB) has been delivering cryopreserved homografts for the treatment of aortic infections to different implanting centres in Europe and elsewhere.

## Material and methods

This paper presents the results of treatment of aortic infection with homografts for diverse indications.

The descending thoracic aortas are recovered during the multi-organ donation (MOD), transferred to the EHB in a cold saline solution (+4°C), decontaminated in a triple antibiotic cocktail for up to 48 hours and cryopreserved using the 10% of dimethyl sulfoxide (DMSO) in Hank’s 199 solution. They are cryopreserved by a *control-rate-freezing* of 1°C/minute down to -40°C and 5°C/min down to -150°C, then stored in liquid nitrogen vapors at a temperature ≤-135°C up to five years. The tissues are delivered to the implanting surgeon on basis of the clinical indication and the state of emergency.

## Preliminary results

Over 20 years, 2,705 arterial segments were delivered for implantation, of which 430 thoracic aortas. 68% of those aortic segments were used for infection related problems (60% for replacement of the infected prosthetic material and 8% for native aortas, mainly for mycotic aneurysms). About 30% of the aortic homografts were used for aortic surgery for non-infectious problems, such as aortic coarctation, hypoplastic arch, malignant infiltration of arterial tree, heart transplantation, prosthetic graft thrombosis or trauma of native arteries. The follow-up of these implanted homografts is in course.

## Conclusion

Aortic homografts are highly resistant against the infection and can offer good long term results for the treatment of infection problems in aortic surgery. The follow-up data will be presented at the Congress venue. Despite very important need for aortic homografts, the offer remains scarce, due to a low donation rate in different countries and a high rate of contaminated tissues during the recovery procedure.

